# TSH-CHECK-1 Test: Diagnostic Accuracy and Potential Application to Initiating Treatment for Hypothyroidism in Patients on Anti-Tuberculosis Drugs

**DOI:** 10.1371/journal.pone.0033704

**Published:** 2012-03-19

**Authors:** Cara S. Kosack, Anne-Laure Page, Leonie T. Van Hulsteijn, Eef G. W. M. Lentjes

**Affiliations:** 1 Médecins sans Frontières, Amsterdam, The Netherlands; 2 Epicentre, Paris, France; 3 Leiden University Medical Center, Leiden, The Netherlands; 4 University Medical Center Utrecht, Utrecht, The Netherlands; McGill University, Canada

## Abstract

**Background:**

Thyroid-stimulating hormone (TSH) promotes expression of thyroid hormones which are essential for metabolism, growth, and development. Second-line drugs to treat tuberculosis (TB) can cause hypothyroidism by suppressing thyroid hormone synthesis. Therefore, TSH levels are routinely measured in TB patients receiving second-line drugs, and thyroxin treatment is initiated where indicated. However, standard TSH tests are technically demanding for many low-resource settings where TB is prevalent; a simple and inexpensive test is urgently needed.

**Methods:**

As a proof of concept study TSH was measured in routinely collected sera at the University Medical Center Utrecht, Netherlands, using the TSH-CHECK-1 (VEDALAB, Alençon, France), a lateral-flow rapid immunochromatographic assay with a TSH cut-off value of 10 µIU/mL, the standard threshold for initiating treatment. These results were compared with TSH levels measured by a reference standard (UniCel DXi 800 imunoassay system, Beckman Coulter, USA). Sensitivity, specificity, and likelihood ratios were then calculated.

**Results:**

A total of 215 serum samples were evaluated: 107 with TSH values <10 µIU/mL and 108 with values ≥10 µIU/mL. TSH-CHECK-1 test sensitivity was found to be 100.0% (95% CI: 96.6–100.0) and specificity was 76.6% (95% CI: 67.5–84.3). Predictive values (PV) were modelled for different levels of prevalence. For a prevalence of 10% and 50%, the positive PV was 32.2% (95% CI: 25.0–39.7%) and 81.1% (95% CI: 75.0–85.5%), respectively; the negative PV was 100% (95% CI: 98.9–100%) and 100% (95% CI: 91.3–100%) respectively.

**Discussion/Conclusions:**

The TSH-CHECK-1 rapid test was practical and simple to perform but difficult to interpret on weak positive results. All sera with TSH≥10 µIU/mL were correctly identified, but the test lacked sufficient specificity. Given its excellent negative PV in this evaluation, the test shows promise for ruling out hypothyroidism. However, so far it appears that samples testing positive with TSH-CHECK-1 would require confirmation using another method.

## Introduction

Multi-drug resistant tuberculosis is a growing problem especially in resource-limited settings and typically requires treatment for at least two years with drugs that can have significant side effects [1]–[5]. Second-line anti-tuberculosis (TB) drugs such as para-aminosalicylic acid, ethionamide, and prothionamide are known to cause hypothyroidism in up to 58% of patients by inhibiting thyroid hormone synthesis through inhibiting the uptake of iodine into thyroid cells and by inhibiting thyroid peroxidise [6]–[13]. These high rates of hypothyroidism have been confirmed in two TB clinics run by Médecins Sans Frontières where about 50% of patients show hypothyroidism after 6 months on these drugs.

Since the symptoms of hypothyroidism can be subtle, the World Health Organization recommends that patients on these second-line TB drugs are screened for hypothyroidism every 6 months, or sooner if symptoms arise [14].

TSH is produced in the adenohypophysis and promotes production of the thyroid hormones T_3_ and T_4_, which are essential for growth and development of the nervous system. Thyroid hormones also affect the synthesis, mobilization and degradation of fat and increase the number of cell surface catecholamine receptors, which in turn affects heart rate. TSH is a marker for thyroid function, and a normal TSH level (<5 µIU/mL) indicates proper thyroid function. Thyroxin is given as therapy for hypothyroidism once a TSH level of 10 µIU/mL is reached [15].

Although monitoring TSH levels in MDR-TB patients is important for managing drug side effects, it can be extremely challenging in low-resource settings: the standard ELISA test requires significant laboratory infrastructure and is relatively costly. A simple, inexpensive test that could substitute for the ELISA assay in identifying patients who need thyroxin treatment (i.e., those with TSH>10 µIU/mL) would greatly improve the monitoring and treatment of hypothyroidism in low-resource settings.

The TSH-CHECK-1 rapid test is a two-band immunochromatographic diagnostic test developed to screen neonates for hypothyroidism; unlike other TSH rapid tests, it is adjusted to detect a TSH level of 10 µIU/mL (rather than 5 µIU/mL, the standard for a diagnosis of hypothyroidism, but not yet requiring treatment). According to the manufacturer, there is a range of ±20% around this cut-off (i.e. 8–12 µIU/mL) when tests are read 10–15 minutes after the addition of sample and diluents. Specificity is claimed to be >95%.

These properties led us to hypothesize that TSH-CHECK-1 could potentially replace the ELISA test in identifying TB patients on second-line therapy and whose TSH levels have reached the point (10 µIU/mL) where thyroxin treatment should begin. We therefore conducted a test-of-concept study using samples from the Netherlands to assess the accuracy of TSH-CHECK-1 against the ELISA reference standard in diagnosing TSH levels above versus below the treatment initiation threshold.

## Methods

### Study design

This study used a nested-case control design to compare the TSH-CHECK-1 rapid test to an immunoassay using chemiluminescent detection technology.

The sample size of 96 positive (TSH levels ≥10 µIU/mL) and 96 negative (TSH levels <10 µIU/mL) samples was calculated based on an estimated 90% sensitivity and 90% specificity with a precision of 6%. Samples were consecutively selected from specimens collected routinely at the Laboratory of Endocrinology in the Department of Clinical Chemistry and Haematology at the University Medical Center Utrecht (UMCU), the Netherlands until the total of 96 ‘positive’ and ‘negative’ samples was reached.

### Test platform and procedure

TSH-CHECK-1 (whole-blood pediatric version, VEDALAB, Alençon, France; reference number 21103) is a lateral-flow rapid immunochromatographic test in cassette format. The device must be stored at 4–30°C and 25 µl of serum/plasma or 50 µl of whole blood are required to perform the test. A negative result (i.e. TSH<10 µIU/mL) is indicated by the presence of a single coloured band in the control zone, while a positive result is denoted by two clearly distinguishable bands. The test is inconclusive if no line appears.

TSH-CHECK-1 tests were carried out according to the manufacturer's instructions on serum within two days of collection. Results were initially read by the technician who performed the test, and then by one subsequent reader. All readers were blinded to the results of the reference test and to one other's readings. Consensus between the latter two readers was considered a definitive result. If there was no consensus, the result from the first reader was used; the dissenting reading was used to assess inter-reader reproducibility.

### Reference method

All samples were analyzed with the UniCel DXi 800 analyzer (Beckman Coulter, Brea, CA, USA) according to the manufacturer's recommendations. The precision of the TSH analysis on the UniCel DXi 800 analyzer is 5–8%.

### Ease of use

Two experienced laboratory technicians reported on the ease of use of the TSH-CHECK-1 test using a standardized questionnaire.

### Statistical analysis

Data was entered into an Excel file and analyzed using Stata 9.0 (Stata Corporation, College Station, Texas, USA). Sensitivity of TSH-CHECK-1 was estimated as the proportion of positive specimens detected among samples with TSH≥10 µIU/mL, as measured by the reference test; specificity was the proportion of negative specimens detected among those with TSH<10 µIU/mL according to the reference test. Likelihood ratios (LR) were calculated using the following formulas:
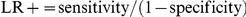
and
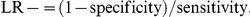
Finally, the positive and negative predictive values were modelled using the estimated sensitivities and specificities for different hypothetical prevalence values. All parameters were estimated with their 95% confidence intervals (CI). Inter-reader reproducibility was measured using the kappa coefficient.

### Ethics statement

The ethics review board of the UMCU examined the study protocol in accordance with the ‘Wet Maatschappelijke Ondersteuning’ (WMO, the Dutch law regulating scientific and medical research) and gave permission to conduct the study without requiring patient consent, since the research used only leftover material (i.e. did not require additional sample collection).

## Results

### Samples

A total of 215 serum samples collected during July–October 2010 were selected for the study: 107 had reference TSH values <10 µIU/mL and 108 had values ≥10 µIU/mL. The median TSH value was 10 µIU/mL (IQR: 2.3–19). The median TSH among patients with reference TSH values <10 µIU/mL was 2.3 µIU/mL (IQR: 1.4–4.3), and among patients with TSH values >10 µIU/mL the median was 19 µIU/mL (IQR: 13–51.6). The mean patient age was 48 years.

### Sensitivity, specificity and predictive values

Compared with the reference standard, sensitivity of the TSH-CHECK-1 test was 100.0% (95% CI: 96.6–100.0) and specificity was 76.6% (95% CI: 67.5–84.3) (Table 1). The positive likelihood ratio (LR+) was 4.3 (95% CI: 3.0–6.0) and the negative likelihood ratio (LR−) was 0.01 (95% CI: 0.0–0.1). Positive and negative predictive values were calculated based on several different hypothetical prevalence levels (Table 2).

**Table 1:** Accuracy of TSH-CHECK-1 test compared against reference standard with >10 µIU/mL as cut-off level for a positive results.

	TSH-CHECK-1		
UniCel DXi 800	Positive	Negative	Total
**Positive**	108	0	108
**Negative**	25	82	107
**Total**	133	82	215

**Table 2:** Positive and negative predictive values based on variable prevalences of TSH level >10 µIU/mL.

Prevalence	Positive Predictive Value (95% CI)	Negative Predictive Value (95% CI)
**5%**	18.4% (13.8–24.1)	100% (99.5–100)
**10%**	32.2% (25.0–39.7)	100% (98.9–100)
**20%**	51.7% (42.9–59.7)	100% (97.7–100)
**30%**	64.7% (56.3–71.7)	100% (96.1–100)
**40%**	74.0% (66.7–79.8)	100% (97.7–100)
**50%**	81.1% (75.0–85.5)	100% (91.3–100)

### Quantitative characterization of TSH-CHECK-1 (Band intensities)

**Figure 1:**
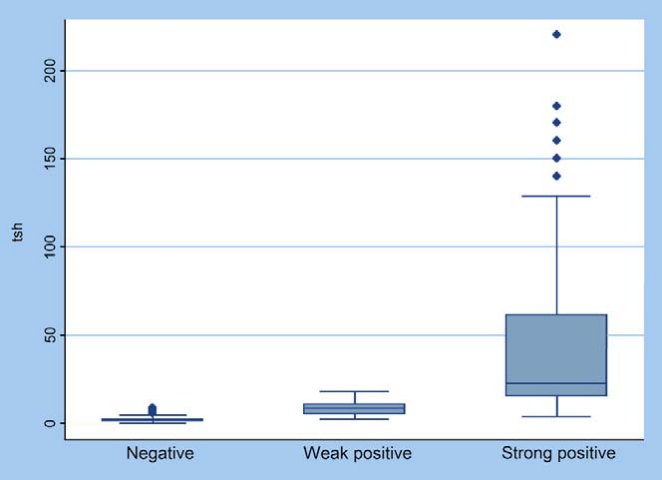
Negative, weak positive, and strong positive test results versus quantitative TSH level (TSH units: µIU/mL).

Negative, faint-positive, and strong-positive results were compared against quantitative TSH levels (Figure 1). Faint positives were found in 36/133 (27.1%) of the positive samples with the TSH-CHECK-1 test when results were read after exactly 15 minutes; these samples showed reference test levels ranging from 2.7 to 12.1 µIU/mL. However, most of these rapid tests turned strongly positive when they were read at 30 minutes; only 5.3% of tests continued to show faint lines.

TSH levels were significantly different between negatives, faint positives, and strong positives (Kruskall Wallis, *P*<0.001).

### Inter-reader reliability

Inter-reader reliability for both positive and negative results was high, with 99.5% agreement between the two readers, and a kappa value of 0.99. For strong band intensities, all readings were identical.

### Ease of use

Based on the results of a questionnaire, we found that laboratory technicians viewed the TSH-CHECK-1 test as practical and simple to perform and the package insert as informative and easy to understand. However, they encountered difficulties in the interpretation of test results, which often appeared as faint but discernible. Faint lines were considered positive since they were clear and distinct, albeit weak. Another problem was that 5/215 (2.3%) of the pipettes delivered with the kit were defective (i.e. leakage) and had to be discarded.

## Discussion

This is, to our knowledge, the first evaluation of diagnostic test accuracy with the TSH-CHECK-1 rapid test (pediatric version). Our aim in this test-of-concept study was to do an initial assessment, in a readily accessible population and setting, of the test's accuracy in distinguishing samples with TSH levels above 10 µIU/mL (the treatment threshold) from those below this level. We found that TSH-CHECK-1 has an excellent sensitivity but only moderate specificity in identifying patients with TSH levels above the cut-off point. Furthermore, 5% (11/215) of patients with TSH levels below the cut-off and within the normal range (<5 µIU/mL) would have been misclassified as eligible for treatment using this test.

This sub-optimal specificity could be due to a variety of factors. Certain clinical factors (e.g. laboratory values) are known to interfere with the TSH-CHECK-1 test and may cause false-positive results; these include high levels of rheumatoid factor or C-reactive protein (CRP), neither of which were measured here. Both should be incorporated into future evaluations to investigate possible cross-reactivity with the TSH-CHECK-1 test.

Technical issues might also play a role, some of which are potentially resolvable. Most false positive results came from tests showing faint lines, raising the notion that a scanning device such as the EASY READER (VEDALAB, France), which has a high-resolution digital camera and can yield quantitative or semi-quantitative data from the test lines' absorbance values, might improve accuracy in interpreting ambiguous tests. We did not use this device in our evaluation, since its relatively high cost and requirement for a dependable supply of electricity limit its usefulness in the lowest-resource settings where a TSH rapid test is most needed.

Another factor that lowers test specificity might be inaccuracies from using the pipetting devices furnished in the rapid test kits; these were less accurate than the built-in calibrated transfer pipettes used in the UniCel DXi 800 imunoassay system, the reference system.

Finally, this evaluation was conducted as a proof of concept study on characterized clinical specimens from a population that is not representative of the population in which the test is intended to be used. It needs to be repeated on consecutive specimens in the target population, i.e. in TB patients receiving second line therapy in a resource-limited setting. Further evaluation should also include a cross-sectional study design, which is considered a more rigorous test of diagnostic accuracy - although the nested case study design used here, when properly applied, is recognized as a reasonably accurate (and much faster and less expensive) initial screen to determine whether a diagnostic test merits further assessment [16], [17].

In conclusion, the TSH-CHECK-1 is an easy-to-use rapid diagnostic test suitable for use in low-resource settings. With an excellent negative likelihood ratio and negative predictive values, the test could be used to rule out hypothyroidism in second-line TB patients. However, samples that test positive on the TSH-CHECK-1 test should be confirmed using another method. Future studies need to be performed to confirm the good performance as a rule-out test in the intended target population, as well as to investigate the potential use of the associated quantitative reader to alleviate the need for a confirmatory test using more sophisticated techniques.
